# A Surprising New Path to Tumor Development

**DOI:** 10.1371/journal.pbio.0030433

**Published:** 2005-11-15

In the long-established model of tumorigenesis, cells acquire specific mutations that disrupt the normal regulatory constraints on unlimited cell division. These mutations allow cells to proliferate at the expense of healthy tissues and develop into tumors. As a result, most cancer treatments use agents that curb tumor growth by inhibiting cell divisions. Surprisingly, a few conditions known to reduce a cell's ability to divide—including genetic traits, some diets, and chemotherapies—seem to increase the chances of tumor occurrence. In a new study, Ganna Bilousova and her colleagues shed new light on this troubling paradox by examining the development of mouse blood cell tumors (leukemias) in contexts that reduce cell division rates.

All circulating blood cells are descendents of progenitor cells that reside in the bone marrow. The ultimate progenitors, called hematopoietic stem cells, give rise to all blood cells by producing more specialized progenitors that initiate distinct lineages, such as the red and white blood cells. Progenitors divide to ensure the constant renewal of blood cells, but their cell divisions must be tightly controlled to avoid generating too many blood cells and increasing the risk of tumors.

Overproliferation of blood cell progenitors seems to underlie chronic myelogenous leukemia (CML) and acute lymphoblastic leukemia (ALL). The blood progenitors of both leukemias harbor a genetic anomaly known as the Philadelphia chromosome. This aberrant joining of Chromosomes 9 and 22 produces an aberrant protein called Bcr-abl, which promotes both cell proliferation and mutations. Either property could account for Bcr-abl's cancer-promoting, or oncogenic, potential. Yet, as the authors show, simply introducing a few blood progenitors carrying the Bcr-abl protein is not enough to produce leukemias in mice. Only when cell divisions are impaired in the mice blood progenitors does the proliferative advantage, and oncogenic potential, of Bcr-abl become obvious.

Their experimental setup mimics the conditions of leukemia onset—in which an oncogenic mutation presumably arises in a single (or a few) blood progenitors—yet eventually allows the cell to supersede its nonmutant counterparts. The authors transplant a mixture of two types of progenitors—some express Bcr-abl and some don't—into mice, and examine their relative contribution to the recipients' blood a few weeks later. (The rodents' own blood progenitors had been previously destroyed by irradiation.) The authors find that whether they slow cell divisions genetically (using stem cells with mutations in key regulators of cell divisions) or chemically (feeding transplanted mice hydroxyurea, a drug commonly used in cancer therapy), the progenitor cells that carry Bcr-abl can overcome the hurdle and produce mature blood cells and, eventually, tumors. In the absence of this challenge, however, Bcr-abl cells barely contribute to the recipients' blood, and far fewer cancers arise. What's more, the authors can quench the oncogenicity of Bcr-abl progenitor cells simply by cotransplanting normal progenitor cells (free of Bcr-abl and able to divide). [Fig pbio-0030433-g001]

**Figure pbio-0030433-g001:**
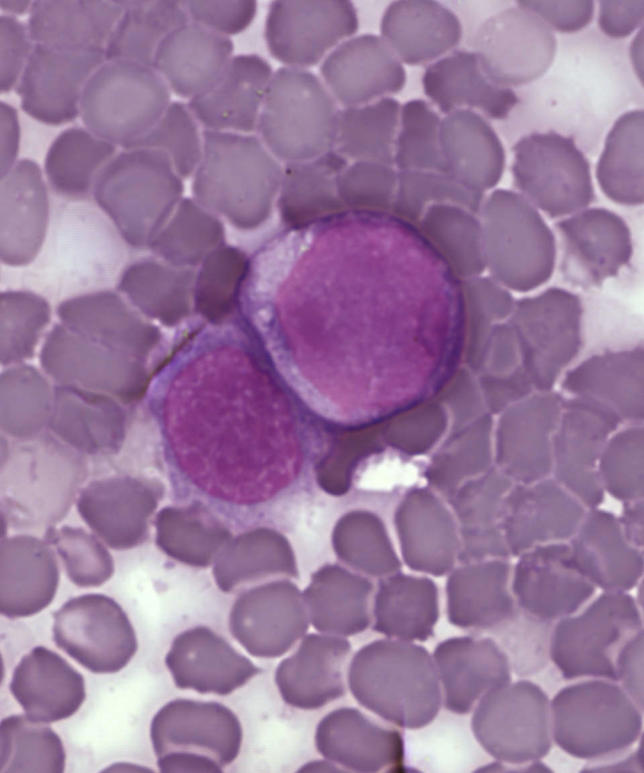
Leukemia cells

The implication of these observations is that nonmutant progenitor cells normally outcompete a Bcr-abl cell arising in a bone marrow niche. But under conditions that impair cell divisions, whether genetic, environmental, or therapeutic, the mutated cell has the advantage and might eventually take over. Overcoming cell division blockers is a tall order that not all oncogenes are expected to fill. Yet mutations in *p53*, an oncogene linked to a wide variety of cancers, confer the same advantage as Bcr-abl in this experimental setting, which suggests that the authors' model might apply to other tumors as well. While much more work needs to be done for the implications of this study to affect people, Bilousova and colleagues speculate that preventing cancer not only involves avoiding exposure to mutation-causing substances, but also a lifestyle that promotes healthy cell division behavior among tissue progenitors. —*Francoise Chanut*

